# Characteristics of emergency patients with markedly elevated D-dimer levels

**DOI:** 10.1038/s41598-020-64853-0

**Published:** 2020-05-08

**Authors:** Ning Tang, Yinyin Pan, Chao Xu, Dengju Li

**Affiliations:** 1 Department of Clinical Laboratory, Tongji Hospital, Tongji Medical College, Huazhong University of Science and Technology, Wuhan, Hubei China; 2 Department of Hematology, Tongji Hospital, Tongji Medical College, Huazhong University of Science and Technology, Wuhan, Hubei China

**Keywords:** Biochemistry, Biomarkers

## Abstract

Background: Markedly elevated D-dimer levels can occur in emergency patients with various clinical situations, and is likely to indicate the presence of coagulopathy, rapid differential diagnosis was crucial for them. Methods: D-dimer was detected in consecutive 813 patients entering the emergency department of our hospital, for the patients with D-dimer levels above 5.0 µg/mL, the final diagnoses and 28-day mortality were confirmed, and the levels of thrombomodulin (TM), thrombin-antithrombin complex (TAT) and plasmin-antiplasmin complex (PAP) on admission were detected. Results: There were 148 emergency patients with D-dimer levels higher than 5.0 µg/mL mainly due to sepsis, malignancy, trauma, venous thromboembolism (VTE), cerebrovascular accident, and so on. Both of the TM and TAT levels among these diagnoses were significantly different (*p* < 0.001). The elevated TM (>13.3 TU/mL) had a predictive value of 96.0% for excluding VTE, and the normal TM had a predictive value of 90.4% for excluding sepsis. The overall 28-day mortality of these patients with D-dimer >5.0 ug/mL was 14.2%, the TAT level on admission was independently associated with 28-day mortality (odds ratio 1.014, 95% CI 1.001–1.027, *P* = 0.030). Conclusions: The medical emergencies associated with markedly elevated D-dimer levels were revealed, specific markers of endothelial dysfunction and thrombin generation measured by automatic analyzer have the potential to distinguish diagnoses and predict outcomes in these patients.

## Introduction

D-dimer is a biomarker of fibrin formation and degradation and has been used routinely for variety of diagnostic purposes, including excluding venous thromboembolism (VTE), determining the optimal duration of anticoagulation in VTE patients, and diagnosing disseminated intravascular coagulation (DIC) *et al*.^1,2^. Emergency patients who present with markedly elevated D-dimer levels are a concern, as they were more likely to have associated coagulopathy^3^. However, markedly elevated D-dimer levels can occur in various clinical situations and is commonly hard to distinguish rapidly^1^, this may due to that as an indirect marker of activation of coagulation as well as fibrinolysis, D-dimer alone can’t indicate the specific state. For instance, with similarly high D-dimer, hyperfibrinolysis is considered to be the major manifestation and therapeutic target of trauma-induced coagulopathy, meanwhile, endothelial dysfunction, excess thrombin generation and fibrinolysis shutdown are the feature of sepsis-induced coagulopathy^4,5^. Hence, more specific coagulation markers may be needed for distinction of these conditions.

In this study, we aimed to investigate the clinical features, diagnoses and outcomes of emergency patients with markedly elevated D-dimer levels on admission; Meanwhile, markers of endothelium, thrombin and plasmin in these patients were detected and evaluated, we assumed that some of these markers might has discriminatory or prognosis value in patients with markedly elevated D-dimer level, i.e. a high possibility of coagulopathy.

## Methods

### Patients

D-dimer was detected in consecutive 813 patients entering the emergency department of our hospital from July 2019 to October 2019. As 3.0–7.0 ug/mL of D-dimer levels had been used as the cutoff of item in several DIC criterias, or to define high risk of VTE^5–7^, we selected the average value of 5.0 µg/mL to define a markedly elevated of D-dimer. A total of 166 (20.4%) patients had D-dimer levels above 5.0 µg/mL (fibrinogen equivalent units, 10 times of the upper normal limit), within 24 hours after admission, and the samples were reserved for further detection.

This study was approved by the Ethics Committee of Tongji Hospital (Wuhan, China), and written informed consent was obtained from all patients or their family members. All methods were carried out in accordance with relevant guidelines and regulations.

Eighteen of the 166 patients were lost to follow-up or died before a diagnosis was confirmed, and were excluded from the study. The remaining 148 patients were all hospitalized, and a retrospective review of their characteristics and outcomes was performed using the hospital’s electronic medical records system, the results of breathing rate, mean arterial pressure, bilirubin and creatinine on admission were recorded to describe multiple organ dysfunctions of each enrolled patient^8^. In addition, in the other 647 patients with D-dimer ≤5.0 ug/mL, 338 (52.2%) were hospitalized, and there were no further investigation on them due to the high rate of lost to follow-up.

### Blood sampling and detection

Blood samples were collected from each patient within 24 hours after admission. Samples were collected into vacuum tubes (BD Vacutainer; Becton Dickinson and Company, Devon, UK) with 3.2% sodium citrate for coagulation tests. The samples for coagulation tests were centrifuged at 2700 × *g* for 10 minutes to obtain platelet-poor plasma, and D-dimer was detected using a STA-R analyzer and STA-Liatest D-Di reagent (Diagnostica Stago, Saint-Denis, France) based on the latex immune turbidimetric assay (reference interval <0.5 µg/mL). As markers of endothelium, thrombin and plasmin, the levels of thrombomodulin (TM), thrombin-antithrombin complex (TAT) and plasmin-antiplasmin complex (PAP) were detected using a HISCL 5000 analyser and original chemiluminescence reagents (SYSMEX, Kobe, Japan), the reference intervals of these three markers were suggested by the manufacturer and had been validated before application in our hospital.

All cases of VTE, aortic dissection, and cerebrovascular accident were confirmed by Doppler ultrasound, computed tomography or magnetic resonance imaging. The diagnosis of sepsis was based on the sepsis 3.0 definition^9^, the presence of infection was confirmed by pathogen detection or comprehensive judgment of our clinician (Dengju Li). Cases of malignancy were confirmed by necessary pathological and imaging examinations.

### Statistical analysis

Quantitative variables are presented as median (interquartile range). Multiple comparisons among biomarkers of each diagnosises were performed using Kruskal-Wallis H test. Between survivors and non-survivors, comparisons of continuous variables were performed using the Mann-Whitney test, and categorical variables were evaluated using Fisher’s exact test or Pearson’s Chi-squared test, where appropriate. Categorical and consecutive variables were evaluated by logistic regression analysis for their ability to predict 28-day mortality. A *P*-value of <0.05 was considered statistically significant. Data were analyzed using SPSS 21.0 for Windows (SPSS Inc., Chicago, IL, USA).

## Results

The 148 consecutive emergency patients with D-dimer levels >5.0 µg/mL included 89 men and 59 women, with a mean age of 56.5 years (range 12–88 years). The primary diagnoses and levels of coagulation biomarkers on admission of these patients are shown in Table 1. Both of the TM and TAT levels among these diagnoses were significantly different (*p* < 0.001).

**Table 1:** Primary diagnoses and levels of specific coagulation biomarkers in emergency patients with D-dimer levels >5.0 µg/mL (n = 148).

Primary diagnosis	Number of Patients (%)	TM (TU/mL)	TAT (ng/mL)	PAP (ng/mL)
**Reference intervals**		**3.8–13.3**	**<4.0**	**<0.8**
Sepsis	36 (24.3)	20.4 (12.9–31.9)	11.6 (5.8–29.8)	2.8 (0.9–5.3)
Malignancy	22 (14.9)	13.4 (8.2–21.0)	11.0 (5.8–23.9)	2.0 (1.2–3.6)
Trauma	20 (13.5)	10.8 (9.3–11.9)	64.1 (21.7–120.0)	3.0 (1.5–10.5)
Venous thromboembolism*	17 (11.5)	10.1 (8.6–12.8)	18.0 (10.6–29.0)	3.1 (1.4–7.2)
cerebrovascular accident	17 (11.5)	19.5 (12.5–30.9)	34.0 (8.5–58.8)	1.9 (1.1–5.2)
Artery dissection	10 (6.8)	15.4 (12.3–17.7)	34.3 (18.7–115.4)	2.6 (1.3–17.9)
Liver cirrhosis	10 (6.8)	16.5 (12.3–25.2)	7.3 (5.0–9.7)	1.9 (0.8–5.3)
Non-infectious inflammation**	8 (5.4)	12.3 (9.5–16.0)	20.7 (11.5–49.1)	1.6 (1.1–1.8)
Others***	8 (5.4)	14.3 (10.1–19.2)	8.4 (6.3–19.5)	1.6 (1.4–3.0)
***P*** **value**		**<0.001**	**<0.001**	**0.44**

*venous thromboembolism was the primary or only diagnosis; **non-infectious inflammation including pancreatitis (n = 4), systemic lupus erythematosus (n = 3) and peptic ulcer (n = 1). *** Other diagnosis including preeclampsia, amniotic fluid embolism, snake bite, using hemocoagulase, nephrotic syndrome, myocardial infarction, and ileus.

Due to the patients with diagnoses of sepsis and VTE had the highest and lowest TM levels, respectively, diagnostic values of TM for sepsis and VTE were evaluated (Table 2). In these emergency patients with D-dimer levels >5.0 µg/mL, the high TM (>13.3 TU/mL) had a sensitivity of 77.8% and a specificity of 58.0% for sepsis diagnosis, and the low TM (<13.3 TU/mL) had a sensitivity of 82.4% and a specificity of 55.0% for VTE diagnosis. Although TAT had the highest levels in trauma patients, and the lowest levels in patients with Liver cirrhosis, these patients commonly had definite medical history, hence the differential value of TAT between trauma and liver cirrhosis didn’t be evaluated. In addition, higher TAT level in patients with artery dissection comparing to those with VTE has been found (*P* = 0.012).

**Table 2:** Diagnostic values of TM for sepsis and VTE in emergency patients with D-dimer levels >5.0 µg/mL.

	Sensitivity (%)	Specificity (%)	Positive predictive value (%)	Negative predictive value (%)
TM > 13.3 TU/mL for sepsis diagnosis	77.8	58.0	36.0	90.4
TM < 13.3 TU/mL for VTE diagnosis	82.4	55.0	19.2	96.0

Correlation analysis showed that D-dimer levels had significant correlations to TAT (*P* = 0.004) and PAP (*P* < 0.001) levels in these emergency patients, the correlation coefficients were 0.236 and 0.558, respectively. No significant correlation was found between D-dimer and TM levels (*P* = 0.570).

The overall 28-day mortality of emergency patients with D-dimer >5.0 ug/mL was 14.2% (21/148 patients). The comparison of age, sex ratio, biomarkers of coagulation and organ function between survivals and non-survivals were showed in Table 3. The non-survivors had significantly higher TM, TAT, total bilirubin and creatinine levels and lower mean arterial pressure on admission than the survivors (*P* < 0.05), these parameters were further evaluated by the multivariable logistic regression analysis (Table 4), and the levels of TAT, mean arterial pressure and total bilirubin were independently associated with 28-day mortality.

**Table 3:** Comparison of markers of coagulation and other organ function on admission between survivals and non-survivals.

	Survivals (n = 127)	Non-survivals (n = 21)	*P* value
Age (years)	60.0 (45.0–69.0)	56.0 (46.5–68.5)	0.752
Sex (Male/Female)	78/49	11/10	0.433
D-dimer (ug/mL)	9.43 (6.77–23.00)	15.24 (8.69–31.56)	0.051
TM (TU/mL)	12.9 (9.7–20.2)	18.6 (12.3–20.2)	0.021
TAT (ng/mL)	15.3 (7.1–35.9)	46.3 (11.7–118.0)	0.005
PAP (ng/mL)	2.02 (1.27–5.37)	3.30 (1.47–8.58)	0.319
Breathing rate	20 (20–21)	20 (19–22)	0.205
Mean arterial pressure (mmHg)	93 (81–101)	87 (66–94)	0.032
Total Bilirubin (umol/L)	11.5 (7.2–16.1)	15.8 (12.4–45.1)	0.035
Creatinine (umol/L)	76 (57–113)	132 (72–186)	0.014

**Table 4:** Multivariate predictive factors of 28-day mortality in patients with markedly elevated D-dimer.

	Multivariate analysis	
	Odds ratio (95% CI)	*P* value
TM	1.017 (0.965–1.071)	0.533
TAT	1.014 (1.001–1.027)	0.030
Mean arterial pressure	0.975 (0.955–0.996)	0.021
Total Bilirubin	1.019 (1.004–1.034)	0.012
Creatinine	1.002 (0.997–1.006)	0.535

## Discussion

Clinicians routinely use D-dimer levels as part of a diagnostic algorithm to exclude a diagnosis of VTE^10^, and the ISTH has also endorsed the role of D-dimer testing in the diagnostic algorithm for DIC^11^. In our study, the emergency patients with D-dimer levels higher than 10 times of the upper normal limit mainly due to sepsis, malignancy, trauma, venous thromboembolism, cerebrovascular accident, and so on. A higher hospitalization rate in these patients were found, comparing to patients with D-dimer ≤5.0 ug/mL (89.2% vs 52.2%). The good correlation between D-dimer and TAT or PAP in our study confirmed the large generations of thrombin and plasmin in these patients, and indicated the existence of coagulopathy.

TM, also called CD141, is an anticoagulant protein constitutively distributed on the surface of vascular endothelial cells. TM binds to thrombin, then this complex activates protein C and forms the major physiological anticoagulant pathway^12^. In sepsis, release of TM from the surface of vascular endothelial cells into the blood circulation partly through proteolytic cleavage by neutrophil elastase^13,14^. Hence, it’s considered to be a biomarker for endothelial cell damage, and that plasma TM levels are elevated in patients with sepsis has been described previously^15^. On the other hand, no association of TM with venous thromboembolism has been found in a former study^16^, also as shown in our study, this may be explained as when VTE is the primary or only diagnosis of a patient rather than being secondary to trauma or infection *et al*., endothelial dysfunction commonly is not the major factor. Hence, it’s reasonable that in our emergency patients with markedly elevated D-dimer, a low or high TM level could be helpful to exclude sepsis or VTE, respectively. In addition, TM had higher on-admission levels in non-survivors compared with survivors, which is consistent with previous studies^17,18^, however, it’s not independently associated with the outcome according to the logistic regression analysis.

As a biomarker related to thrombin generation, TAT had the highest level in trauma patients of our study, that has also been described in previous reports^19,20^. Although it’s not needed to distinguish trauma patient from others by blood testing, TAT might be used for prognosis as it was associated with the outcome and independent of other organ dysfunctions in this study. In addition, as both of VTE and artery dissection could present markedly elevated D-dimer and similar symptoms^21^, patients with artery dissection showed higher TAT level, this indicates more obvious coagulation activation in artery dissection than in VTE and the potential of differential diagnose, and also can be used to explain that why patients with artery dissection often show a DIC-like coagulopathy^22^. However, a study with larger sample size is needed to confirm it. Relatively, as a biomarker of plasmin generation, PAP seemed to be of little differential or prognosis value in patients with markedly elevated D-dimer.

According to these results, it seemed that detection of these specific coagulation markers could support a further explanation of elevated D-dimer mechanistically, moreover, unlike most previous studies, the TM, TAT, and PAP levels were all measured by an automatic analyzer in our study, the shorter turn-around time and simpler methodology ensure that these biomarkers can be used for emergency case.

This study had some limitations. The TM, TAT and PAP levels of patients with D-dimer less than 5.0 ug/mL had not been detected and evaluated. In addition, we defined the markedly elevated D-dimer as higher than 10 times of the upper normal limit (5.0 ug/mL) according to previous studies^5–7^, this definition might still be arbitrary.

Nevertheless, the current results revealed that medical emergencies commonly associated with markedly elevated D-dimer levels, which were correlated with more productions of thrombin and plasmin, and indicated the risk of coagulopathy. Plasma TM and TAT levels measured by automatic analyzer have the potential to distinguish diagnoses and predict outcomes in these emergency patients.
